# Supplementary material for: Cerebrospinal Fluid Alpha-Synuclein Improves the Differentiation between Dementia with Lewy Bodies and Alzheimer’s Disease in Clinical Practice
Source: Int J Mol Sci. 2022 Nov 4;23(21):13488. doi: 10.3390/ijms232113488 (PMC9654229; doi:10.3390/ijms232113488)
Supplement: Supplementary file 1 [file ijms-23-13488-s001.zip › ijms-2000921-supplementary.pdf]

Supplementary Materials:

Figure S1. Flow chart of the study

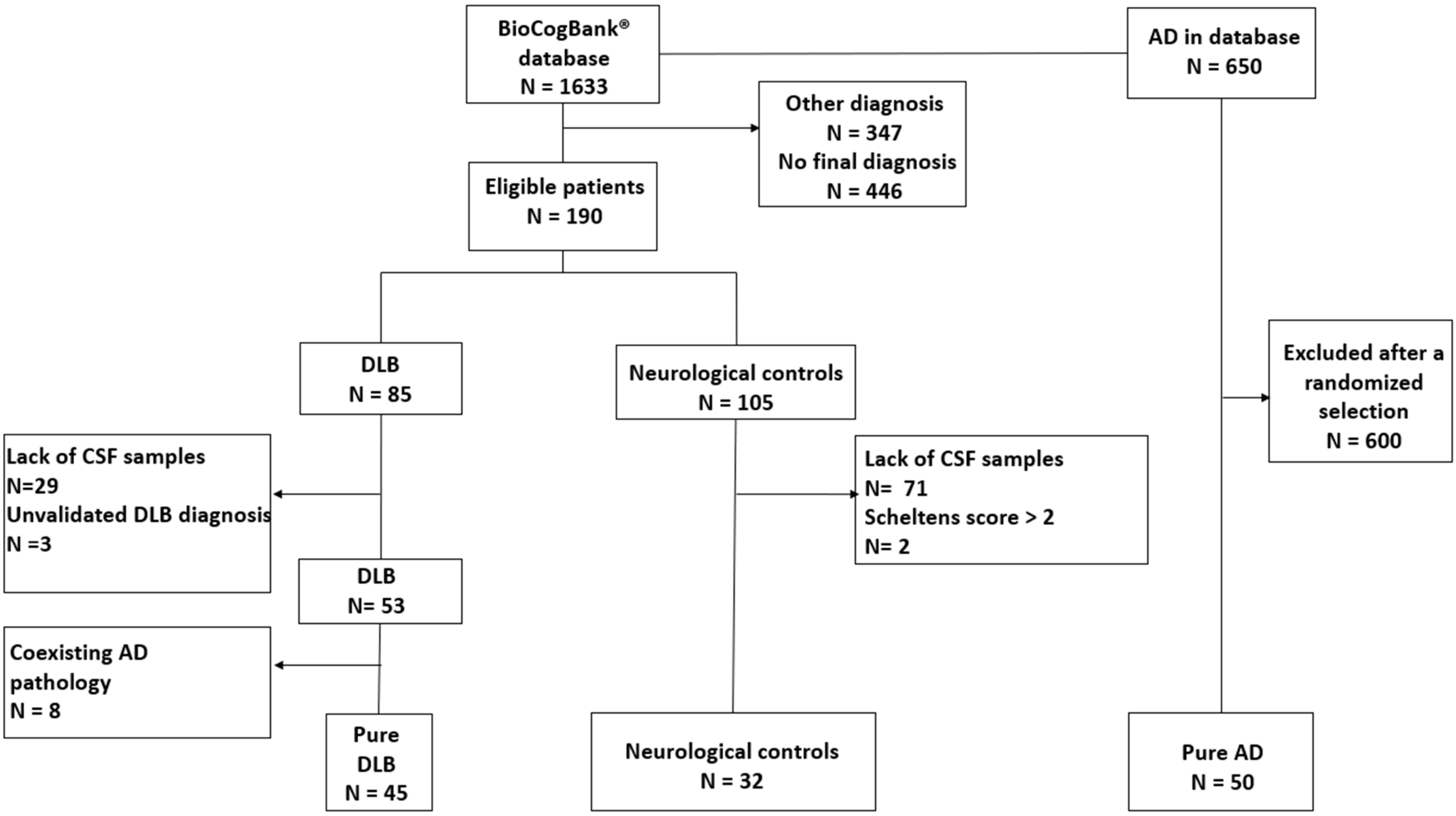

AD: Alzheimer's disease, CSF: Cerebrospinal Fluid, DLB Dementia with Lewy bodies.

Table S1. Cut-offs of CSF biomarkers measures for defining ATN profile

Date / Cut-off	Aβ-42 (pg/mL)	Total Tau (pg/mL)	Phosphorylated Tau (pg/mL)	Aβ-40/Aβ-42 Ratio
Before Dec 2012	< 500	> 300	> 65	Not performed
Dec 2012 to May 2016	< 815	> 300	> 58	> 15,5
May 2016 to May 2018	< 730	> 340	> 58	> 13
Since May 2018	< 860	> 225	> 22	> 12

Aβ: β-amyloid peptide

Brief comment regarding previously published studies in this field

The available body of evidence is characterized by high heterogeneity, preventing from drawing reliable conclusions. In early reports, the diagnostic criteria used to define Alzheimer's disease (AD) or Dementia with Lewy bodies (DLB) were inconsistent, the study samples were of limited size (ranging from 6 to 67 patients with DLB). Most of these analyses were performed using non-standardized sandwich enzyme-linked immunosorbent assay (ELISA) to measure the CSF α-syn concentrations. Some of them were designed to quantify disease-specific isoforms but not the cerebrospinal fluid (CSF) total amounts of the protein. These differences explain the major discrepancies between their conclusions: several authors did not report any difference between groups [7–11], one study highlighted increased CSF levels in DLB [12] whereas most of the published studies found lower CSF α-syn in DLB [13–21]. Consistent with our results, most studies that compared AD and DLB CSF α-syn levels observed increased CSF α-syn levels in AD compared to DLB [14–21] while some of them described higher levels in DLB [7,12] or no difference [8–10,13]. Yet, unlike in most of the former studies [13–17] we carefully reviewed all the diagnoses of the included participants according to the last validated criteria. More specifically, unlike in Bousiges et al. [21] we only included patients with pure DLB i.e. CSF AT(N) profile excluding concomitant AD pathology. We also included clinical and neuroimaging data in the diagnostic process.
